# Evaluating the implementation fidelity to a successful nurse-led model (INTERCARE) which reduced nursing home unplanned hospitalisations

**DOI:** 10.1186/s12913-023-09146-8

**Published:** 2023-02-09

**Authors:** Raphaëlle A. Guerbaai, Sabina DeGeest, Lori L. Popejoy, Michael Simon, Nathalie I. H. Wellens, Kris Denhaerynck, Franziska Zúñiga

**Affiliations:** 1 Institute of Nursing Science, Department Public Health, Faculty of Medicine, University of Basel, Bernoullistrasse 28, 4056 Basel, Switzerland; 2 Academic Centre for Nursing and Midwifery, Department of Public Health and Primary Care, KU Leuven, Leuven, Belgium; 3 Sinclair School of Nursing, University of Missouri, Columbia, United States of America; 4 La Source School of Nursing, HES-SO University of Applied Sciences and Arts Western Switzerland, Lausanne, Switzerland

**Keywords:** Nurse-led models of care, Implementation science, Implementation fidelity, Advance-care planning, Complex intervention

## Abstract

**Background:**

Implementation fidelity assesses the degree to which an intervention is delivered as it should be. Fidelity helps to determine if the outcome(s) of an intervention are attributed to the intervention itself or to a failure of its implementation. Little is known about how fidelity impacts the intended outcome(s) and what elements or moderators can affect the fidelity trajectory over time. We exemplify the meaning of implementation fidelity with INTERCARE, a nurse-led care model that was implemented in eleven Swiss nursing homes (NHs) and showed effectiveness in reducing unplanned hospital transfers. INTERCARE comprises six core elements, including advance care planning and tools to support inter- and interprofessional communication, which were introduced with carefully developed implementation strategies.

**Methods:**

A mixed-methods convergent/triangulation design was used to investigate the influence of implementation fidelity on unplanned transfers. A fidelity questionnaire measuring the degree of fidelity to INTERCARE’s core components was fielded at four time points in the participating NHs. Two-monthly meetings were conducted with NHs (September 2018-January 2020) and structured notes were used to determine moderators affecting fidelity (e.g., participant responsiveness). We used the fidelity scores and generalized linear mixed models to analyze the quantitative data. The Framework method was used for the qualitative analysis. The quantitative and qualitative findings were integrated using triangulation.

**Results:**

A higher overall fidelity score showed a decreasing rate of unplanned hospital transfers post-intervention (OR: 0.65 (CI = 0.43–0.99), p = 0.047). A higher fidelity score to advance care planning was associated with lower unplanned transfers (OR = 0.24 (CI 0.13–0.44), p = < 0.001) and a lower fidelity score for communication tools (e.g., ISBAR) to higher rates in unplanned transfers (OR = 1.69 (CI 1.30–2.19), p = < 0.003). In-house physicians with a collaborative approach and staff’s perceived need for nurses working in extended roles, were important moderators to achieve and sustain high fidelity.

**Conclusion:**

Implementation fidelity is challenging to measure and report, especially in complex interventions, yet is crucial to better understand how such interventions may be tailored for scale-up. This study provides both a detailed description of how fidelity can be measured and which ingredients highly contributed to reducing unplanned NH transfers.

**Trial registration:**

The INTERCARE study was registered at clinicaltrials.gov Protocol Record NCT03590470.

**Supplementary Information:**

The online version contains supplementary material available at 10.1186/s12913-023-09146-8.

## Background

Decreasing unplanned hospital transfers (i.e., emergency department visits with or without ensuing hospitalisation) is a goal for many nursing homes (NH) [1] since these transfers are associated with an increase in negative outcomes for residents such as falls, delirium, or nosocomial infections, and are costly for health systems [2, 3]. In the last decades, models of care, quality improvement (QI) programs and individual interventions have been developed and implemented in NHs with the goal of reducing unplanned or avoidable hospitalisations from NHs [4–10]. The Improving INTERprofessionalCARE for better resident outcomes – the INTERCARE model, comprised six evidence-based core components and was developed within a theory-based implementation science study which relied on a thorough contextual analysis, continuous stakeholder involvement, evidence-based interventions and the development of implementation strategies targeting specific barriers and facilitators [11–13]. The INTERCARE model was successful in decreasing unplanned hospitalisations of NH residents [14] as well as cost-effective [15] and highly acceptable from the NH staff’s perspective [16].

Differentiating implementation effectiveness from clinical effectiveness is essential to distinguish intervention failure (the intervention is unsuccessful) from implementation failure (flawed implementation) [17]. To evaluate implementation effectiveness, implementation outcomes, defined as “effects of deliberate and purposive actions to implement new treatments, practices, and services” p. 65 [17] are measured and reported [18]. One outcome is implementation fidelity, defined as the degree to which a program is delivered as it was intended to be by its developers [17]. Implementation science frameworks (e.g., RE-AIM) specifically guide researchers to plan for and report implementation science outcomes such as fidelity [19] to better understand which program components are correctly adhered to [20, 21] and to describe the supportive factors for the implementation, such as the implementation strategies used. Implementation fidelity is considered as a mediator between intervention components and clinical outcome(s), and helps to unravel the reasons behind an intervention’s success or (partial) failure [20]. Additionally, implementation fidelity provides relevant information to help tailor both intervention components and implementation strategies, and it informs scale-up and translation of interventions to different settings [20, 22].

Few multi-component interventions or studies specifically referred to as quality improvement (QI) studies conducted in NHs examine the relationship between implementation fidelity and its influence on clinical outcomes (e.g., unplanned hospital transfers) [23]. Only 3% of NH QI studies report fidelity, however QI guidelines do not emphasize reporting this outcome [24]. The Interventions to Reduce Acute Care Transfers (INTERACT) quality improvement (QI) program was the first initiative to develop a bundle of tools that can be implemented in NHs to drive staff clinical reasoning and communication with the goal of reducing avoidable hospitalisations [4]. The degree of implementation of the INTERACT was evaluated and found that the increased usage – a way of measuring fidelity – of the INTERACT package was associated with a greater reduction of hospitalisations [4], but underlined the need to further assess implementation fidelity as factors which contribute to decreasing hospital transfers remain unclear [23]. Moreover, uncertainty remains regarding the variability of results on the outcome of reducing hospital transfers between studies that used similar components, as some had a positive effect and reduced unplanned hospitalisations from NHs [25–27], whilst others did not [7, 8, 28]. There is a need to understand how fidelity to core components enabled the success or partial failure of the intervention as this can inform adaptations for further implementation in other settings. Implementation fidelity is supported by tailored implementation strategies, which facilitate the implementation and sustainment of an intervention and are unequivocal to reach implementation fidelity. However, the evaluation of implementation strategies and the impact these had on the INTERCARE study is beyond the scope of this article and will be the focus of the forthcoming process evaluation. To our knowledge, studies evaluating the implementation fidelity to nurse-led care models implemented in NHs to reduce hospitalisations have not been conducted or reported to date.

## Methods

### Aim

This study aims to:

1. Evaluate the degree of implementation fidelity to INTERCARE and to each of the core components over time, and the relationship fidelity has with reducing unplanned transfers (Quantitative aim).
2. Explore factors that might affect the implementation fidelity to the core components of the INTERCARE model (Qualitative aim).
3. Gain an understanding of which factors influenced the fidelity trajectory of core components over time (Mixed-method aim).

### Design

**Fig. 1:**
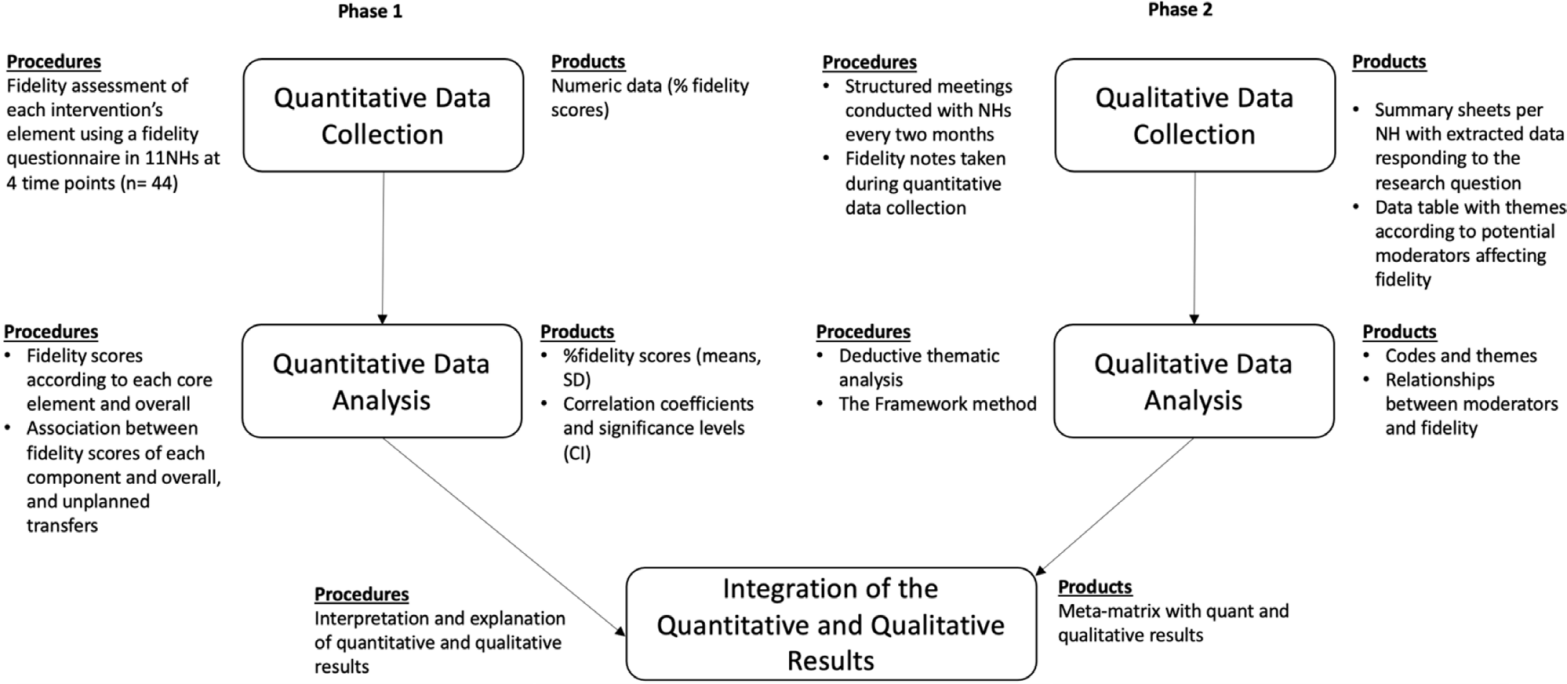
Representation of mixed methods convergent/triangulation design

This study is part of a larger hybrid type 2 implementation science study which applied a non-randomized stepped-wedge design to roll-out a nurse-led model of care - INTERCARE in eleven Swiss-German NHs. The overall objective of INTERCARE was to reduce unplanned transfers from NHs [11]. For the study described in this paper, a mixed-methods convergent/triangulation design was used to investigate the influence that implementation fidelity to the model of care had on unplanned transfers [29]. This mixed methods approach was chosen as thorough integration of findings from both quantitative and qualitative methods achieve a more comprehensive answer for the study aims. Figure 1 graphically illustrates the study design.

### Conceptual framework

**Fig. 2:**
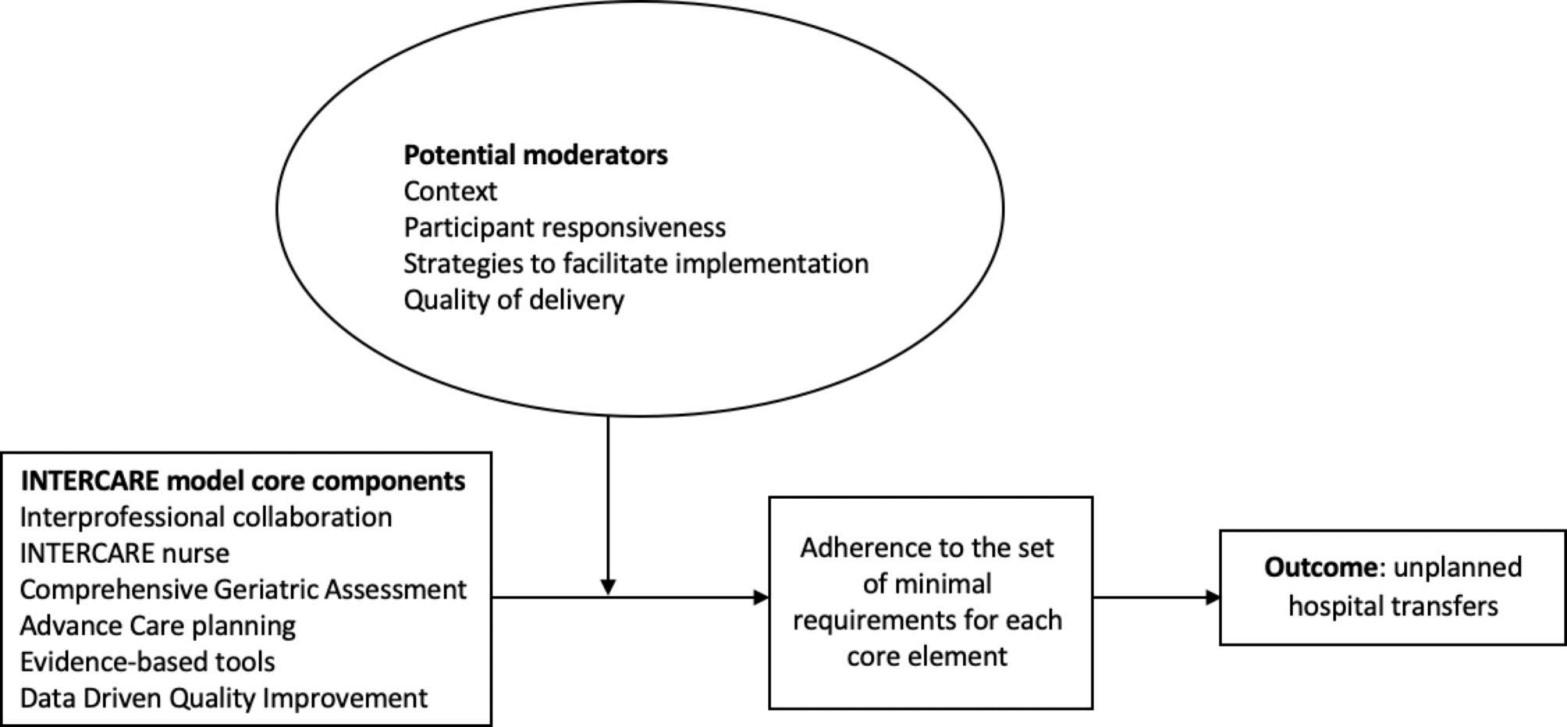
The Conceptual Framework for Implementation Fidelity adjusted for the INTERCARE study, adapted from Carroll et al. [31]

A modified version of the Conceptual Framework for Implementation Fidelity developed by Carroll et al. [30] was used as the overarching frame for the implementation fidelity study of INTERCARE. It was adapted as not all concepts of Carroll et al’s framework were measured and reported here, such as the evaluation of outcomes and the component analysis as described by Carroll et al. [30]. This framework helped us conceptually think about the evaluation of implementation fidelity and how fidelity could impact INTERCARE’s main outcome. Additionally, the potential moderators suggested by Carroll et al. were fitting to what we could expect from the data sources we collected to measure fidelity but also other implementation outcomes (Fig. 2). The degree of implementation fidelity for this study is defined as adherence to the set of minimal requirements defining each core element of the INTERCARE intervention.

### Description of INTERCARE

#### Intervention components

A full description of the INTERCARE core components including their minimal requirements can be found in Supplementary file 1. Each component was defined according to minimal requirements which needed to be fulfilled for the components to be considered implemented. A set of peripheral components were also defined for each core element, these could be adapted by each NH as local adaptations improve the fit of the intervention to different contexts, thus increasing successful implementation [19]. In brief, INTERCARE’s six core components included (1) strengthening of **interprofessional collaboration** through the development of internal structures and processes to facilitate communication between physicians and NH staff; (2) an **INTERCARE nurse** specifically appointed or hired in each NH having fulfilled advanced training in geriatrics with at least a registered nurse’s diploma and three years professional experience in NHs [13]; (3) conducting a **comprehensive geriatric assessment** (**CGA)** of the residents initiated by INTERCARE nurses when a change in condition was observed; (4) the introduction of **evidenced-based tools** from the INTERACT program, including STOP&WATCH to guide the detection and communication about changes in residents’ condition at time of observance, ISBAR for structured communication between nurses and physicians, and an acute care transfer reflection tool adapted from the INTERACT’s Acute Care Transfer Tool [4] to analyse reasons for unplanned transfers;[6] (5) **advance care planning (ACP)** to help NHs initiate early discussions about end of life care and document residents’ wishes; and (6) **data-driven quality improvement** feedback to the NHs, to identify potential areas for improvement (e.g., unplanned transfers).

#### Implementation strategies

To support the implementation of the above-mentioned core components, implementation strategies were developed based on a prior contextual analysis to address barriers and facilitators [12]. In summary, the implementation strategies developed for INTERCARE focused on the introduction to the INTERCARE model and regular exchanges with NH managers, project managers and INTERCARE nurses. The INTERCARE nurses’ role was supported by an ongoing training and learning program and bi-weekly phone calls for coaching of this new role. Audit and feedback were provided to NHs for benchmarking and internal quality development. A full description of the implementation strategies can be found in Supplementary file 2.

### Quantitative phase

#### Data collection and procedures

The quantitative data for the present study was collected at four time points for each of the participating NHs during the INTERCARE study. A fidelity questionnaire was developed containing all core components and their minimal requirements, each minimal requirement was considered as a fidelity item in the questionnaire (Supplementary file 3). All fidelity items remained the same for the four measurement points.

Measurement points included baseline (T0), six-months after intervention start (T1), twelve-months after intervention start (T2), and nine-months after the intervention ended (T3).

At measurement point T0, each NH fidelity questionnaire was filled out by the research team based on information the team had previously collected during leadership meetings with the eleven NHs concerning the intervention components which were already in place (i.e., advance care planning (ACP)). For measurement points T1 and T2, the INTERCARE study coordinator completed the fidelity questionnaire during a one-hour telephone interview with the INTERCARE nurse in each NH. Any missing results were discussed during the next NH leadership meeting which occurred bi-monthly, to avoid any missing data and provide clarification if needed. For measurement T3, the questionnaires were completed by the NHs during an online post-study meeting, held nine months after the study ended. The final data was comprised of four fidelity questionnaires for each NH (total of forty-four fidelity questionnaires), that were merged into one dataset and were coded with a randomly attributed NH number, then stored on a secured server.

### Variables and measurement

#### Fidelity

Each core component was defined prior to the intervention roll-out, there was a minimum number of requirements that had to be fulfilled for the intervention core element to be considered adhered to. To measure and evaluate fidelity for the component of “evidence-based tools”, the component was divided into separate components (STOP&WATCH tool and ISBAR tool), each with their own set of minimal requirements. The final set of minimal requirements were locked after the last NH started with INTERCARE; thus, no modifications were made after the last NH implemented INTERCARE. This set of minimal requirements formed a questionnaire used to address the delivery of each core component. Each minimal requirement was rated as either “yes” equating to a score of 1 or “no” equating to a score of 0 (Supplementary file 3).

For the component of ACP, two minimal requirements were measured in the fidelity questionnaires and three minimal requirements where integrated into a case report form used to collect overall residents’ medical information. These three requirements questioned clarification of resident care goals in three areas; were [1] Do not Resuscitate Status, a [2] Do Not Hospitalize Status and [3] preference for antibiotics in a palliative care situation, had been clarified with residents.

An *overall fidelity score* (i.e., percentage of minimal requirements overall delivered as intended) per time point and NH was calculated. The *fidelity scores* for each core component of the INTERCARE model: Interprofessional collaboration, INTERCARE nurse, comprehensive geriatric assessment, ACP, evidenced-based tools (divided into two separate components, the STOP&WATCH tool [6], and the ISBAR tool [6]) and data driven quality improvement. For each of the above-mentioned components, the *fidelity scores* were calculated as overall mean fidelity of content scores (i.e., percentage of minimal requirements per core components delivered as intended). Levels of fidelity were interpreted as previously reported in the literature [31, 32], with 80–100% adherence interpreted as ‘high’ fidelity, 51–79% as ‘moderate’ and 0–50% as ‘low’ fidelity. INTERCARE was considered successfully adhered to in each NH if the overall fidelity score was > 80%, and this threshold was also applied to individual core components (e.g., if > 80% was achieved for the core element advance care planning, we considered it successfully adhered to).

#### Unplanned hospitalisations

Unplanned hospitalisations were measured as the main outcome of the INTERCARE study and described elsewhere [14]. Residents with informed consent hospitalisation data (Transfer date, type of transfer (planned/unplanned), date of discharge and preliminary diagnosis) were collected throughout the study. *Unplanned hospitalisations* were defined as a transfer from the NH to a hospital (including emergency department visits) for an unplanned reason (e.g., fall), including ED admissions and excluding psychiatry referrals.

### Statistical analysis

Analyses were performed using SAS 9.4 (SAS Institute, Cary, NC) and R 3.5.2 (Eggshell Igloo) [33], with dplyr [34] and tidyverse [35]. We predicted the probability of daily unplanned transfer status (yes or no) by 6-months fidelity, using generalized linear mixed models. Each NH received a random intercept and repeated observations were nested within residents. Generalized estimation equations accounted for clustering of the repeated measures and random variable. The same models were computed with the fidelity scores for each separate core component of INTERCARE as described above, to assess their individual relationship to unplanned transfers.

### Qualitative phase

#### Data collection and procedures

Qualitative data was collected during bi-monthly meetings organized with each participating NH, whereby leadership teams, INTERCARE nurses and other professions were invited to attend (e.g., NH’s responsible physician). The INTERCARE study group led the meetings and it was comprised of the INTERCARE study leader, the study coordinator and two research assistants. The meetings were mainly open discussions moderated by the INTERCARE research group, based on the same structure for every meeting. Each INTERCARE core element was discussed with regards to how the implementation was going, whether some issues were experienced and how the implementation of each core element was facilitated with NHs. The NHs usually described specific situations to illustrate how core components were handled and the research coordinator took notes and summarized the discussion points. A spreadsheet structured according to the core elements, was used for each NH to collect information during the meetings by the research coordinator and checked and supplemented by the study leader. The meetings were held in Swiss German. The spreadsheets were translated via an online software system, DeepL®, into English to fasten the translation process and make it easier to extract the data. The translated spreadsheets were checked for accuracy by two native speakers present at every meeting, including the research coordinator who was the responsible person for completing the spreadsheets.

### Data analysis

Deductive thematic analysis was supported by The Framework Method to extract meaningful data from the leadership meeting notes [36]. This procedure was chosen due to its flexibility to analyse qualitative notes and for its rigorous procedure which is easy to follow and apply to large datasets [37]. Prior to starting with the analysis, four NHs were selected by the first and last author based on their varying fidelity trajectories from the quantitative data as a basis to develop a code book of thematic codes to subsequently apply to the following seven NHs. First, the initial spreadsheet for each NH containing all the data from the leadership meetings was reduced to contain only the pertinent information relating to implementation fidelity to the core components. The potentials moderators from the Conceptual Framework for Implementation Fidelity, namely: context, participant responsiveness, NH strategies to facilitate implementation and quality of delivery were used as the overarching themes to deductively select pertinent information, as the amount of data was overwhelming for eleven NHs and the moderators captured the information we wanted to report. Additional factors or results will be integrated into the process evaluation paper. A definition of each moderator can be found in Table 1. Two researchers (RAG, first and FZU, last author) trained in qualitative methods screened each reduced NH’s spreadsheet to extract data according to the moderators. Separate NH data tables were generated with information relating to each core component corresponding to the above moderators. RAG and FZU discussed and defined emerging codes based on three NH data tables, for each core component. The reliability of the codes was discussed with a third reviewer (project coordinator who was present at each meeting and took notes). The code book containing the moderators and associated codes can be found in Supplementary file 4.

**Table 1:** Definition of the moderating factors identified for the INTERCARE study

Potential Moderator^1^	Definition
Context	Surrounding systems, structures, cultures of NHs and concurrent events.
Participant responsiveness to the delivery of the core components of INTERCARE	Refers to how well the NH staff, leadership, physicians, respond to or are engaged by each core component. It can include judgment about relevance and outcomes of the components. Low participant responsiveness implies that the less enthusiastic participants (leadership, NH staff, INTERCARE nurse, physicians) are about a component, the less likely the component is to be implemented properly and fully.
Strategies to facilitate implementation of the core component	Supporting strategies developed by the NHs, which include standardized written procedures, in-house training, guidelines and actions to enhance buy-in and optimize the implementation of the core components.
Quality of the core component’s delivery	Description of methods used to appropriately deliver the core components to achieve the intended goal or generate results.

^1^Definitions were adapted from Carroll et al., and tailored to the INTERCARE study

### Integration

Integration of quantitative and qualitative data occurred at an interpretation level using the triangulation methodology [38]. A meta-matrix was developed comparing the quantitative results to the qualitative themes (Table 2).

**Table 2:** Triangulation of quantitative and qualitative results: convergence of findings

Core Element	Fidelity Trends from The Quantitative Findings	Moderating Factors for Fidelity from The Qualitative Findings	Triangulation
Interprofessional Collaboration	- Moderate fidelity at baseline - Maintained high fidelity during intervention/sustained post-intervention	**Context** The working practices embedded in NHs prior to the implementation of INTERCARE, such as physician preferences regarding communication and cantonal policies, hindered or facilitated fidelity to this core component. As such, NHs working with responsible physicians had a better basis to develop the interprofessional collaboration. **Participant responsiveness** NHs enrolled had a communication structure in place prior to the implementation of INTERCARE, which explains moderate fidelity at baseline. NHs made changes in communication structures (such as strengthened reports) at the start of the implementation of INTERCARE which helped develop and strengthen communication between nurses, physicians, and/or therapists and which led to improved collaborations. High fidelity was achieved and sustained after six months after implementation of INTERCARE due to changes in practices which enhanced perception of professional roles, (i.e., nurses felt listened to by physicians and respected). In NHs working with a general practitioner model^2^, some general practitioners showed resistance to change and collaboration with NH staff was not improved. **Quality of delivery** NHs described how they optimized the communication process to facilitate communication achieving desired results (i.e., collaborative approach to treatment plans).	Convergence
INTERCARE Nurse	- Low to high fidelity achieved within the first six months post implementation of INTERCARE - Maintained high fidelity during intervention/ sustained post-intervention	**Context** INTERCARE nurses worked in different positions with different roles prior to endorsing the INTERCARE role such as working as registered nurse in a team or as unit leader in some NHs. **Participant responsiveness** The INTERCARE nurse’s role developed rapidly in the NHs with INTERCARE nurses expressing growing self-efficacy and self-confidence, clear responsibilities and boundaries. Although it didn’t hinder reaching high fidelity within the first six months of the study, clarifications as to when the INTERCARE nurse was needed and feeling overwhelmed due to uncertainty of this new role was acknowledged by the INTERCARE nurses. It was clear that the INTERCARE nurse’s role was perceived as a relief for NH staff in challenging situations, and widely accepted by NH staff who responded well to being coached and empowered. Overall, it was acknowledged that there was a need for the INTERCARE nurse’s role. Across the various NHs, the INTERCARE nurses fulfilled a number of outcomes depending on the NH needs, including interval trainings, improving their coaching skills helping NHs work independently and leading expert discussions. **Strategies to facilitate implementation** NHs introduced the INTERCARE study and INTERCARE nurse’s role as possibilities to better prepare for future challenges. Promoting the INTERCARE nurse’s visibility and part taking in unit activities. **Quality of delivery** Achieving high fidelity can be explained by high commitment to the INTERCARE role. INTERCARE nurses put effort into being present on units, engaging in unit rounds, combining the INTERCARE role with unit leader role for some of the INTERCARE nurses already working in that role. Physicians invested into training INTERCARE nurses, and having physicians in the background helped the role grow, even though this was not necessarily the case for all NHs and not a minimal requirement for the fidelity measurement. In NHs with an external GP model (i.e., working with external GPs), GPs could not engage in coaching, this didn’t seem to affect reaching high fidelity for those NHs.	Convergence
Comprehensive Geriatric Assessment	- Moderate fidelity reached at six months and maintained until twelve months. Moderate fidelity decreased to low fidelity post-implementation of INTERCARE	**Participant responsiveness** Comprehensive geriatric assessment (CGA) was perceived as important by INTERCARE nurses and aspects of this elements were conducted (e.g., clinical assessment with lung auscultation, pain assessment) but not always reviewed and follow-up accordingly, which explained the moderate fidelity during the intervention period. While all NHs assessed with which assessment instruments they worked and where change was needed, the INTERCARE study required them to support care team in integrating the 5 dimensions of CGA in daily practice. This proved challenging concerning financial and psychosocial aspects, which explains that not all minimal requirements could be fulfilled. **Strategies to facilitate implementation** NHs purposely postponed the introduction of CGA and related tasks to invest in the implementation of other components in view of prioritizing resources and reducing burden. For the elements of CGA which could be implemented, support given on a peer-to-peer basis helped foster putting knowledge into practice (i.e., interpretation of information gained from an assessment). **Quality of delivery** INTERCARE nurses which demonstrated willingness to implement aspects of CGA reported a lack of “fertile soil” although performing certain clinical tasks (e.g., lung auscultation) prevented some unplanned transfers from occurring. Also, when assessment instruments were used, lack of follow-up was observed, which didn’t improve during the intervention period.	Convergence
Advance care planning (ACP)	- Moderate fidelity was reached progressively during the intervention period, and continued to increase in the post-intervention period reaching a high degree of fidelity nine months after INTERCARE ended.	**Context** In most NHs the development and implementation of ACP was slow as time was needed to think and develop clear NH structures and processes needed for the implementation of ACP. Some NHs already had a basis or ACP in practice and INTERCARE helped NHs to strengthen prior practices and facilitate culture change or help NHs to further develop their needs (this was further supported through the COVID-19 pandemic). One NH acknowledged that ACP as a growing theme in the heath sector helped support the introduction of ACP overall. **Participant responsiveness** Medical engagement for ACP varied across NHs, with ACP being challenging with external general practitioners as opposed to having a responsible physician participating in or overlooking the implementation of ACP in the NH. NHs were able to demonstrate that they had a system in place to anticipate issues occurring during out-of-hours to better address critical situations. **Strategies to facilitate implementation** NHs developed documents to help facilitate the implementation of ACP (e.g., emergency plan).	Convergence
Communication tool: Stop & watch	- High fidelity was achieved during the first six months of implementation but decreased continuously thereafter to moderate fidelity.	**Context** The first NH to start with the implementation reported lacking in information and materials regarding the tools which slowed down their implementation at the start. **Participant responsiveness** The STOP&WATCH (S&W) tool helped nursing aides to pass on information proudly and NHs reported high staff buy in at the start. Effort was needed to help nursing aides to think for themselves and to not be afraid of using the tool (fear to commit a mistake). This tool was used in different ways, for handover to structure thoughts, but also extended to night teams and activity staff. For some NHs, S&W helped initiate a culture change and follow-up was requested. Although advantages were seen, some barriers were noted, such as difficulty in starting with this tool due to unclarity regarding its usage, misconceptions and how to handle feedback once a tool has been completed. Some INTERCARE nurses and physicians were uncertain as to how to use the STOP&WATCH tool within the nursing team, therefore the minimal requirements were not met. **Strategies to facilitate implementation** NHs trained champions to help introduce the S&W tool and progressively implemented tools on all units. Displaying the S&W tools enhanced their visibility and helped facilitate implementation.	Convergence
Communication tool: ISBAR		**Participant responsiveness** Implementation of the ISBAR tool support decision making and NH staff attitudes toward ISBAR were very positive. They felt valued and able to communicate on an equal level with physicians with professionalism. ISBAR helped with changes in communication practices such as better prepared and structured phone calls between physicians and nurses. Vital signs and better documented and communicated which many physicians missed until the introduction of ISBAR. The utilization of ISBAR was extended to email and unit visits, so that any email correspondence was structured according to the ISBAR tool and contained information in a structured way and unit visits could be prepared in advance. **Strategies to facilitate implementation** NHs trained champions to help introduce the ISBAR tool and progressively implemented tools on all units.	Convergence
Data driven quality improvement	- High fidelity was reached at six months after implementation of INTERCARE and sustained after INTERCARE ended	**Participant responsiveness** NHs discussed benchmarking reports and quality improvement charts to identify areas for improvement. Some NHs identified specific procedures in place such as regular meetings to work on quality improvement. NHs identified some challenges such as high staff turnover which made it difficult to implement assessments or consistently work on a theme, however this did not seem to affect fidelity to this element. Once main requirement was to use a Plan-Do-Act-Cycle to work on a theme in order to improve and some NHs were not able to use it or mis-used it for other issues not quality related. **Strategies to facilitate implementation** The NHs were supported by the study leader to better understand how to tackle quality management and NHs demonstrated that different channels (i.e., variety of meetings between different professionals) were available to discuss topics.	Convergence

^1^In Switzerland, nursing homes are required to abide to cantonal policies and these regulate with which medical model nursing homes work with. A general practitioner model refers to nursing homes working with general practitioners as opposed to having a responsible physician for the nursing home.

## Results

### Quantitative results

Eleven NHs were included in the INTERCARE study [11, 14]. The NHs and resident characteristics can be found in a prior publication [14].

#### Implementation fidelity over time

**Fig. 3:**
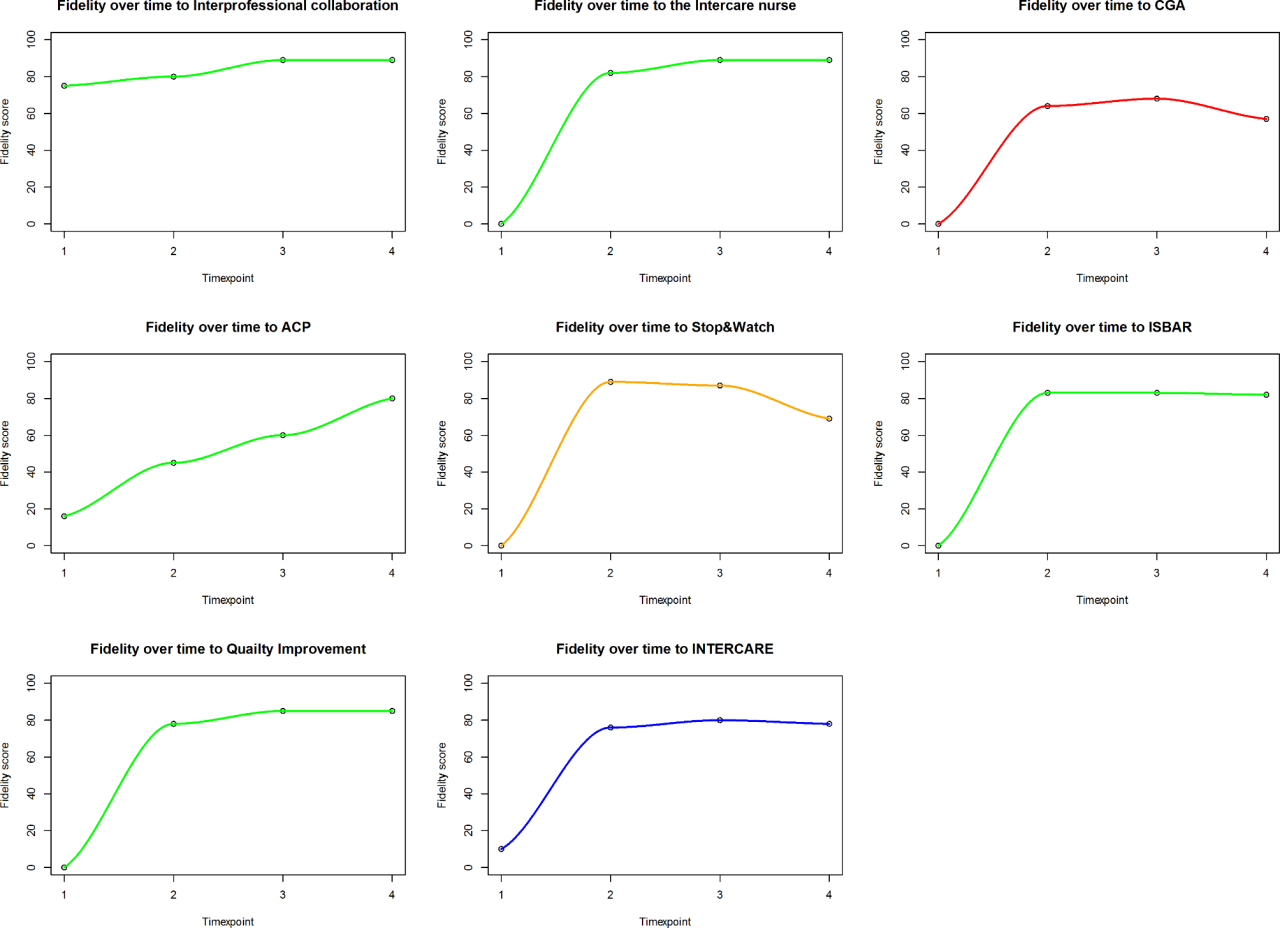
The fidelity trajectory for each core element over time and INTERCARE overall Legend: x axis: study time point, 1 = T0; 2 = T1; 3 = T2, 4 = T3 ; y axis: fidelity score with 0 = 0% and 100 = 100% Core components in green reached a high-fidelity score at T4, the one in Orange reached a high-fidelity score at 12 months but dropped and in red the one component which failed to achieve high fidelity. Overall fidelity to INTERCARE is represented in blue and depicts high fidelity reached at 6 months and sustained thereafter

**Fig. 4:**
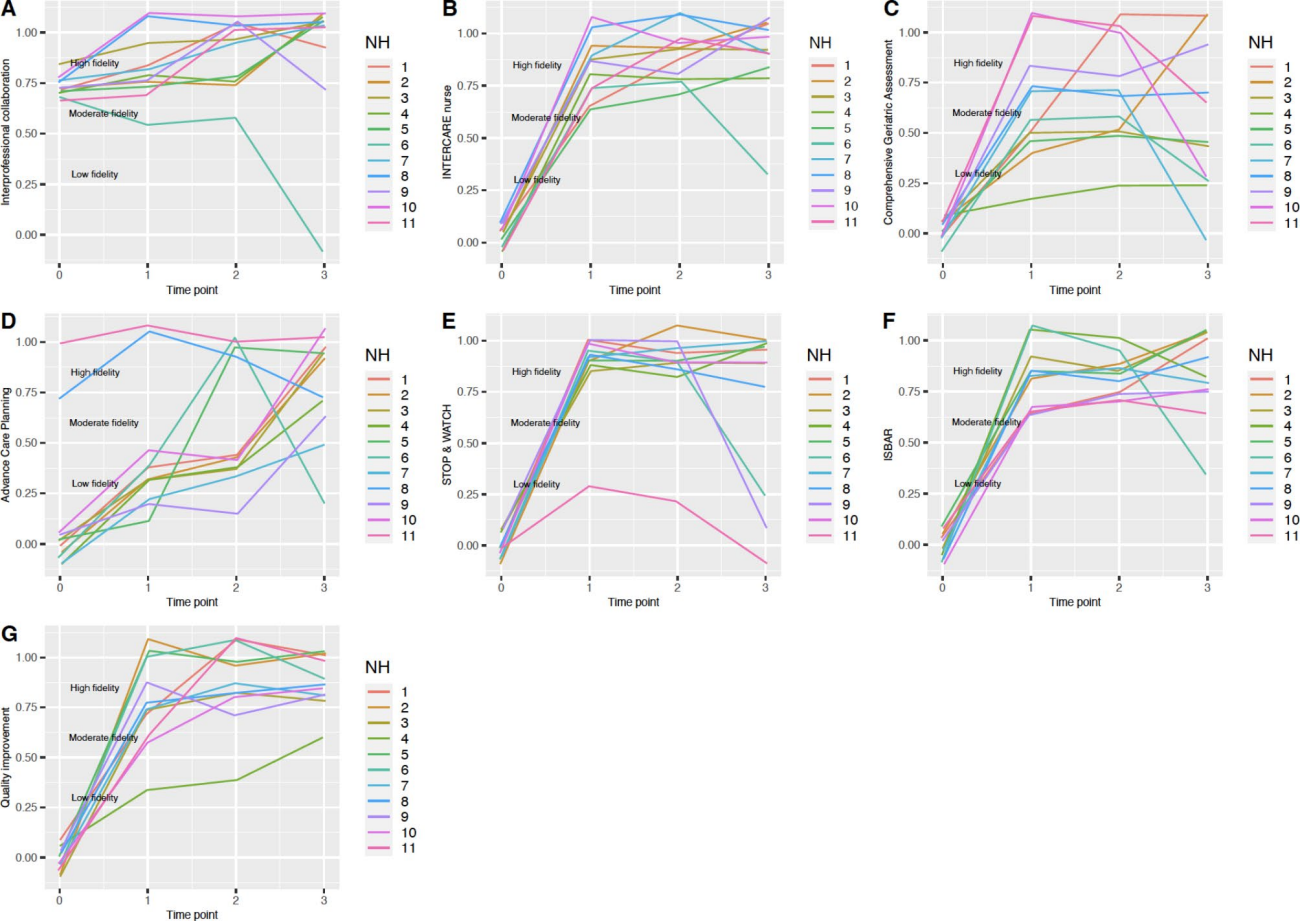
Separate fidelity trajectories per nursing home and per INTERCARE core component (A-G). Jittering function was applied to increase space between the data points to help better visualize the similar NH trajectories Legend: x axis: study time point, 0 = T0; 1 = T1; 1 = T2, 3 = T3; y axis: fidelity score with 0 = 0% and 1.00 = 100%; High fidelity: >0.8%, Moderate fidelity: 0.51–0.79%, Low fidelity: <0.50%

Figure 3 illustrates the trend in implementation fidelity at the four time points measured during the INTERCARE study (baseline until 9 months after the intervention ended) for each of the core components. Figure 4 depicts the fidelity trajectory separately for each of the core components in each of the 11 NHs included. NHs reached high fidelity within the first six months of the intervention for the following components: the INTERCARE nurse, STOP&WATCH, ISBAR and for data driven quality improvement. Only two NHs reached high fidelity for the components of ACP, comprehensive geriatric assessment and interprofessional collaboration within the first six months, however these components were already implemented as part of regular NH routine in these NHs. The other NHs engaged to change their regular practice and to introduce and implement these three components according to the INTERCARE protocol. Two components, comprehensive geriatric assessment and STOP&WATCH, decreased in fidelity over time. For comprehensive geriatric assessment, four NHs decreased in implementation fidelity overtime. For STOP&WATCH, eight NHs decreased in implementation fidelity overtime.

#### The fidelity score and time interaction effect

**Fig. 5:**
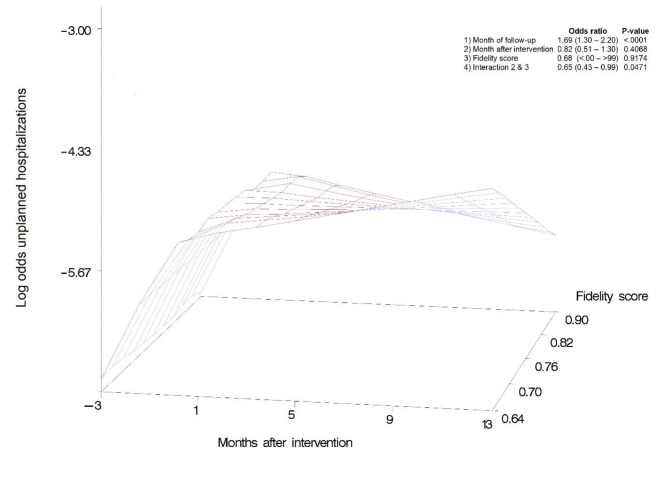
Three-dimensional representation of the fidelity score-dependent intervention effect

Analysis of the relationship between fidelity scores and unplanned transfers showed a significant interaction effect between fidelity and time (OR: 0.65 (CI = 0.43–0.99), p = 0.047). As illustrated in Fig. 5, higher fidelity scores showed a decreasing rate of unplanned transfers post-intervention, while for the lower scores, the unplanned transfers kept increasing. The relationship between time and unplanned transfers was different for varying fidelity scores, and a reduction of unplanned transfers over time was found for higher fidelity scores only. Concretely, for a fidelity score of 0.6, at start of the intervention, the probability of an unplanned transfer was 0.5%, while it was 1% one year later. However, for a fidelity score of 0.9, these numbers were 0.4% and 0.2%, respectively. ACP scores did not show an interaction effect, however higher scores were associated with lower unplanned transfers (OR = 0.24 (CI 0.13–0.44), p = < 0.001). Likewise, lower fidelity scores for the STOP&WATCH tool showed higher rates in unplanned transfers (OR = 1.69 (CI 1.30–2.19), p = < 0.003). The degree of fidelity to each of the following components, namely, the INTERCARE nurse, ISBAR, data driven quality improvement, interprofessional collaboration and comprehensive geriatric assessment components did not have statistically significant relationships to unplanned transfers.

### Qualitative results

Context, participant responsiveness, NH strategies to facilitate implementation and quality of delivery were retained as the potential moderating factors to the fidelity trajectories observed in the participating NHs. Each moderating factor was used to explain how they played a part in modifying the fidelity trajectories of each of INTERCARE’s core components, as well as explaining the variability in implementation fidelity in the NHs.

#### Context

NHs were not uniform in their capacity to implement INTERCARE. Contextual factors that prepared them to implement INTERCARE were crucial moderators as these impacted the fidelity trajectories due to structures and processes which were already in place prior to the implementation of INTERCARE (e.g., already using ACP). NHs working with a responsible physician had a stronger structure in place to work with. This helped NHs adhere to the minimal requirements of the different components (e.g., interprofessional collaboration, ACP), compared with NHs working with multiple general practitioners, where more effort was needed to get them accustomed to internal changes these brought after implementation. Contextual variability existed between NHs working with different physician models. NHs with responsible NH physicians described having their support (e.g., during medical ward rounds) whereas NHs working with different physicians had to accommodate to their preferences which made it more challenging to develop a strong communication process for interprofessional collaboration (e.g., some physicians preferred email over in-person meetings, difficulties to contact them).

NHs with a responsible physician had - to some extent – began working with aspects of core components (i.e., ACP) prior to implementing INTERCARE, which helped them further progress with the implementation of additional requirements of the INTERCARE model. Similarly, working with nurses in extended roles prior to implementing INTERCARE facilitated adherence to the INTERCARE nurse component but also supported the adherence to the other core components, increasing the degree of implementation fidelity in those NHs. Furthermore, variability was seen between NHs working with responsible NH physicians and INTERCARE nurses with prior experience in an extended role, and NHs working with general practitioners and INTERCARE nurses without prior experience. NHs in the latter category struggled to achieve high fidelity to ACP as this component demanded more time, a level of skill and knowledge as well as experience and support, which was often provided by responsible physicians for the NHs managing to achieve high fidelity and sustain it.

In NH units with high staff turnover, difficulties to satisfy the minimal requirements and reach high fidelity for core components were raised, as consistent workflow was challenging to establish (i.e., to sustain utilization of STOP&WATCH and to steadily work on quality improvement).

#### Participant responsiveness

Participant responsiveness had a direct impact on the degree of implementation fidelity over time. NH staff, NH leadership and physicians (some initial resistance was perceived from external general practitioners) saw the need for a role which could support NH staff and act as a “resource person” such as the INTERCARE nurse. There was a perceived need for improving and strengthening the quality of information handed over to various professionals, contributing to the attractiveness for STOP&WATCH and ISBAR tools which both received very positive attitudes mainly from nursing aides (STOP&WATCH) and from nurses and physicians (ISBAR), during the first six months after implementation. Unclear processes regarding the handling of STOP&WATCH impeded the usage affecting fidelity to this component [39]. ACP is an example of a component which requires changes at several levels (policy level, NH level, individual level) and requires time for NHs to tackle the associated requirements. Better understanding of NH needs and feasibility regarding comprehensive geriatric assessment could help tailor the component to make sense for participants and improve fidelity to this component.

#### Nursing home strategies to facilitate implementation

Prior to the implementation of INTERCARE, the research group developed implementation strategies based on a prior contextual analysis to help facilitate and sustain the introduction of the INTERCARE model [11, 40] (see Supplementary file 2). While these strategies targeted NH leadership teams and INTERCARE nurses, NHs developed their own approaches to facilitate the implementation of the core components in the care teams and among other professionals affected to help achieve high fidelity. Contextual issues such as high staff turnover were challenging for the NHs which led them to purposely postpone the implementation of certain core components and to choose to prioritize which components to implement first to reduce burden. NHs worked on positively promoting INTERCARE as a whole and increasing visibility, encouraging INTERCARE nurses to be accessible on the units and making the evidence-based tools (STOP&WATCH, ISBAR) visible and accessible to all NH staff. Units identified champions to help implement the tools and other aspects of some components (ACP, data driven quality improvement). Other strategies included peer to peer support to help putting knowledge into practice (for comprehensive geriatric assessment) or the development of channels to help discuss quality issues between different professionals.

#### Quality of delivery

NHs described a variety of ways to ensure the core components were appropriately implemented, which contributed to the NHs reaching high fidelity for most components. A collaborative approach was used to discuss the processes needed to fully implement the core components and identify which steps were needed. NHs were highly committed to implementing the core components of INTERCARE as intended. Some differences between NHs concerning the development of the INTERCARE nurse’s role based on the NHs’ vision and resources influenced the quality of how the intervention component of the INTERCARE nurse was implemented. For instance, some INTERCARE nurses were able to conduct training sessions whereas others were not due to the nurses’ perceived knowledge limits and lack of time required to organize and prepare such trainings. Unclear processes hindered the quality of how the STOP&WATCH tools were implemented, as tools were filled out, but NH staff did not know how to react once a tool had been filled out. This was very similar for comprehensive geriatric assessment, as lack of follow-up, after an assessment was performed, weakened the quality of implementation and lead to it gradually being relinquished for most NHs. A full meta-matrix with the triangulation results can be found in Table 2.

## Discussion

This study sought to better understand how the degree of implementation fidelity to a nurse-led model of care “INTERCARE” evolved over time and how it impacted the reduction of unplanned transfers. INTERCARE was implemented in eleven NHs following implementation science methods. We found that most core components of INTERCARE reached and sustained high fidelity throughout the intervention period and even extended post-intervention. Across the time span of the project, higher fidelity scores to the INTERCARE model overall lowered the risk of an unplanned hospital transfer. When modeled separately, higher fidelity scores to ACP and to ISBAR and STOP&WATCH tools were associated with a decrease in unplanned hospital transfers. Moreover, the qualitative findings helped to understand how the moderating factors selected from the Conceptual Framework for Implementation Fidelity [30] affected the fidelity trajectory over time and varied between NHs. The physician model, prior knowledge and practice of core components (context), the perceived need of the intervention element (participant responsiveness), the use of key persons/champions (NH strategies to facilitate implementation) and commitment to the core components (quality of delivery), were key moderators for implementation fidelity to INTERCARE.

### Advance care planning (ACP)

ACP is considered as a key component of nurse-led models of care and has contributed to reducing hospitalisations in previous studies [41, 42]. For INTERCARE, the ACP element’s main attributes were to record residents’ wishes to three questions (whether they wished for a transfer in an acute situation, for cardio-pulmonary resuscitation, and for antibiotics in palliative care). Most NHs included in the INTERCARE study struggled to reach high fidelity to ACP in the first year of the intervention (despite national Swiss guidelines, only two NHs were working with ACP prior to the start of INTERCARE). Specifically, INTERCARE nurses found it challenging to find the time to initiate conversations with residents and their relatives and to involve the resident’s physician in these conversations partly due to working with many different general practitioners. In other models working with ACP such as The Missouri Quality Initiative, which demonstrated a positive effect in reducing hospitalisations, interdisciplinary teams were used that included licensed social workers to work with the advanced practice registered nurses to facilitate end of life and goals of care discussions [41, 43]. According to other studies, ACP is highly relevant for NHs and contributed to reducing unplanned and avoidable transfers [44] but uptake remains low in NHs [45]. Collingridge Morre and colleagues comment upon the fact that fidelity to palliative care interventions including ACP is underreported but brought to light a number of barriers and facilitators which could be addressed to possibly increase uptake and embeddedness of ACP in NHs [46]. Addressing high staff turnover, supporting the development of skills such as increasing palliative care knowledge within care teams and for NHs to have access to external support, can greatly enhance the success of ACP interventions [46]. Our findings suggest that nurses working in extended roles such as INTERCARE nurses can drive the implementation of ACP and are well-equipped to conduct discussions, however implementing ACP in NHs requires processes and it takes time until these are fully embedded into daily practice. Therefore, considering implementing specifically trained staff such as social workers in NHs who can manage the complex nature of ACP could help to support the implementation of ACP and obtain implementation fidelity sooner.

### ISBAR and STOP&WATCH tools

We found that higher fidelity to the ISBAR communication tool and STOP&WATCH tool reduced unplanned transfers and this is consistent with Huckfeldt et al’s findings [21]. The implementation of these tools was thoroughly discussed with all NHs which participated in INTERCARE and were adapted according to their needs. The INTERCARE study coordinator helped guide the implementation of these tools through bi-weekly phone calls where barriers to implementation were identified and strategies to overcome the issues were discussed. Moreover, the implementation was supported through availability of the tools in different formats such as laminated pockets cards, to help staff become acquainted with the tools and facilitate higher fidelity. These tools were also found to be highly acceptable and feasible to implement [39]. Despite achieving high fidelity at the start of the INTERCARE project, the fidelity scores for STOP&WATCH decreased after a year. Indeed, in its current form, the STOP&WATCH tool was very attractive to NH staff, in particular nursing aides, as they could report resident changes in condition and were given more credibility. Nonetheless, in most NHs this tool stopped being used despite achieving high fidelity during the first six months. This was most likely because there was not a clear process describing nursing actions needed after the tool was handed over to the appropriate professional. This proved to be too complex to manage and could not be implemented successfully during the entirety of the INTERCARE study. This is similar to findings from the pilot BIRCH program where the STOP&WATCH tool was seldom used despite initial enthusiasm [47]. NHs achieved high implementation fidelity for ISBAR and sustained it after the intervention ended. NHs reported adapting the usage of the ISBAR tool and extending it to an email template to improve both oral and written communication between nurses and physicians but written communication as well.

### Potential moderators

The potential moderators chosen from Carrol et al’s framework guided the deductive thematic extraction of data to explain the core component’s fidelity trajectory [30]. This approach was used in other studies examining fidelity in different contexts [22, 48]. Using these moderators as overarching themes is helpful to gain an overall understanding of some of the mechanisms impacting fidelity. Further investigation as to how potential moderators impact one another deserves more attention with specific attention to any adaptations made throughout the implementation. The list of potential moderators proposed by Carrol et al’s framework could be extended to include NH specific moderators [22].

### Measurement of fidelity

Very few studies conducted in NHs have measured or reported on the degree of fidelity of a complex intervention. Thus, efficient ways of measuring fidelity in the NH setting are lacking [21, 23], although approaches have been developed and reported to guide fidelity assessments, these are limited to NH behavioral interventions [49, 50]. There are a number of barriers to the evaluation of implementation fidelity which may explain why few NH studies measure and report fidelity. First, complex interventions and their components have to be clearly defined by the study team to be able to accurately measure fidelity [51]. Poorly defined core components lead to imprecise measurement of components and a lack of clarity regarding which are being faithfully replicated [20, 22]. We used a pragmatic method based on the resources we had, as no evidence-based psychometric tools to measure fidelity have been developed, adapted and tested in a variety of fields [52] unlike for other implementation outcomes (e.g., acceptability, feasibility) [53]. We did this by defining minimal requirements for each core component of the INTERCARE model and asking the INTERCARE nurses if the minimal requirements were fulfilled or not by means of a questionnaire. This method may not be sensitive enough to thoroughly measure implementation fidelity.

Fidelity can be defined and measured according to concepts of “content”, “coverage”, “frequency” and “duration” [30] or “adherence”, “dose” and “reach”, and sometimes a mixture of these [17, 54]. For instance, we convened to measure fidelity by evaluating whether a minimal requirement was performed as it should, thus assessing only “adherence” and not measuring any of the other above-mentioned concepts, which could oversimplify how we measured fidelity, which is a consistent struggle for other studies as well [51]. Conducting direct observations in the NHs or asking the INTERCARE nurses to write a daily diary could have possibly contributed to strengthening the measurement of fidelity through the measurement of dose.

We did not measure fidelity in a way which captured specific adaptations to the implementation of each core element and thus cannot report whether small adaptations occurred during the implementation phase. Some practical challenges to measuring fidelity have been described. Choosing the right unit of analysis for fidelity measurement is challenging in complex NH interventions as different components target different levels (i.e., unit level and individual level). Also, internal validity threats can jeopardize data collection as focus groups aiming to collect information about fidelity can become part of the intervention and modify participant behavior [51]. These challenges can complicate operationalization of fidelity especially when resources are limited. Although thresholds for fidelity to categorize high, moderate and low fidelity are consistent across studies, no « consensus » has been agreed upon to define what constitutes high, moderate or low fidelity and what these categories mean for complex interventions (e.g., what does high fidelity mean for a given component?) [51]. For instance, for INTERCARE, some minimal requirements could have possibly been given higher weight compared to others and thus deemed more important to fulfill to reach high fidelity. In this study, all core components were given the same importance, but did not have the same number of minimal requirements.

### Limitations

Overall our sample size was rather small with eleven NHs participating, which can lead to little variability between NHs and is a challenge in similar studies [55]. Due to time and resource constraints, only the INTERCARE nurses were asked to complete the fidelity questionnaires during a phone call which could have induced self-reporting bias and potentially recall bias [56]. We used self-reported data to measure the STOP&WATCH and ISBAR tool use as it was not feasible to measure their usage in detail at the facility level without the tools being in an electronic health record or onsite quantification of tool use.

## Conclusion

Evaluation of implementation fidelity is needed to better understand how a complex intervention reaches clinical effectiveness. It helps practitioners, policymakers and other key stakeholders to understand if an intervention is being replicated as it should and to understand why comparable programs vary in their results. This study showed that high overall implementation fidelity to INTERCARE was necessary to reduce unplanned hospitalisations from NHs and it adds confidence that the decrease in unplanned transfers is attributed to INTERCARE. Furthermore, high implementation fidelity to ACP and communication tools directly contribute to decreasing unplanned transfers. Exploring moderating factors can help understand how implementation fidelity can change over time and expose which aspects need further development or better strategies to increase implementation fidelity as well as its sustainability after the research intervention has ended. Length of study is important to reflect fidelity changes over time. Future research needs to focus on more robust methods to measure and analyze fidelity in complex interventions conducted in NHs.

## Electronic supplementary material

Below is the link to the electronic supplementary material.

Supplementary Material 1

Supplementary Material 2

Supplementary Material 3

Supplementary Material 4

## Data Availability

While the data upon which this study’s findings are based are available from participating NHs, restrictions apply to their availability. I.e., they were used under license for the current study, and are not publicly available. However, data directly relevant to our findings are available from the corresponding author upon reasonable request and with permission from the participating NHs.

## List of abbreviations

ACP: advance care planning
CGA: comprehensive geriatric assessment
INTERACT: Interventions to Reduce Acute Care Transfers
INTERCARE: Improving INTERprofessionalCARE for better resident outcomes
NH: nursing homes
QI: quality improvement
