# Supplementary material for: Evaluating the implementation fidelity to a successful nurse-led model (INTERCARE) which reduced nursing home unplanned hospitalisations
Source: BMC Health Serv Res. 2023 Feb 9;23:138. doi: 10.1186/s12913-023-09146-8 (PMC9910256; doi:10.1186/s12913-023-09146-8)
Supplement: Supplementary file 1 — Supplementary Material 1 [file 12913_2023_9146_MOESM1_ESM.docx]

**Additional file 1–** Core and peripheral elements of the INTERCARE model

The following table provides the list of core components of the nurse-led model of care, which correspond to the minimal requirements and the peripheral components, which can be tailored to each nursing home (NH).

| Core elements | Additional information for minimal requirements | Peripheral |
| --- | --- | --- |
| **Interprofessional collaboration** | | |
| A structure in place to facilitate interprofessional communication (e.g., meetings) between at least two different professions. | Each NH^[[1]](#footnote-1)^ is free to decide how communication between different professions may occur, for instance regular team meetings or unit rounds. | Number of structures in place and who involved in the communication structures. |
| Noticing a resident issue and liaising with the relevant health care professional to establish the residents’ care goal. |  |  |
| Interpretation of assessment results and formulation of a resident care plan in collaboration with a member of the health care team. |  |  |
| The INTERCARE nurse supports the communication process between physicians and health care staff. | This can occur by having a prior discussion (in person or phone call) with the care staff before they contact the physician. The INTERCARE nurse might guide the care staff to think through a situation and think about potential questions the physician may ask. |  |
| **INTERCARE nurse** | | |
| According to the INTERCARE nurse’s skills and expertise residents are assessed in acute situations, when called by a member of the care team. |  | Range of educational backgrounds: RN^[[2]](#footnote-2)^, BSN^[[3]](#footnote-3)^, MSN^[[4]](#footnote-4)^, MAS^[[5]](#footnote-5)^, HöFa I and II^[[6]](#footnote-6)^  Number of patients the INTERCARE nurse is responsible for in each NH.  Number of units the INTERCARE nurse works on in the NH.  The way and frequency in which the educational sessions are delivered |
| The INTERCARE nurse provides coaching to care staff on daily resident bedside needs. | The INTERCARE nurse supports care staff by assisting, guiding or advising them during bedside care, for instance helping staff to communicate with a resident showing aggression |  |
| The INTERCARE nurse plans educational sessions with care staff regularly. | The INTERCARE nurse can choose a topic of interest to help care staff improve their competencies and knowledge. These educational sessions can be conducted as formal presentations or by the bedside depending on the topic chosen. The INTERCARE nurse can use their own experience to help care staff manage often occurring difficult situations. |  |
| The INTERCARE nurse drives team reflections for each reflection tool filled in. | The INTERCARE nurse plans informal team meetings to reflect and learn from each reflection tool, with the staff present at the time of the acute situation leading to the hospitalization. |  |
| The INTERCARE nurse must have 3 years-experience in long-term-care. |  |  |
| A position of 60% minimum per 80 beds for which the INTERCARE nurses are responsible for. |  |  |
| **Comprehensive geriatric assessment (CGA)** |  |  |
| The INTERCARE nurse collaborates with the leadership and/or interprofessional team to discuss and define which assessment instrument they work with, for each of the 5 CGA dimensions in their institution, within the first 6 months of the implementation of the model. | The CGA includes the following dimensions:   - Physical dimension - Functional dimension - Social dimension - Economic dimension - Mental dimension | Each INTERCARE nurse is free to define how involved they are and the degree of responsibility they have for each dimension.  Any care staff can be involved in the 5 dimensions of the CGA, corresponding to their degree of training and experience |
| The INTERCARE nurse’s role is clearly defined with regards to their input in the 5 dimensions of CGA. |  |  |
| The INTERCARE nurse is involved and supports the care team in integrating the 5 dimensions of CGA in daily practice. | Provides information and guidance to the care team about the 5 different dimensions and can suggest how each dimension can be assessed and evaluated. |  |
| The INTERCARE nurse ensures that residents and relatives are involved in the decision-making process. |  |  |
| **Advance care planning (ACP)** | | |
| For every newly admitted resident, the following points must be documented in the residents’ records:   - - Do not resuscitate order   - Do not hospitalize order   - Use of antibiotics |  | Presence of physician during initial conversation and subsequent conversations with residents/relatives.  Degree of involvement of the NH staff in ACP discussions  The INTERCARE nurse is in charge of ensuring that every question is clarified with residents and relatives.  The INTERCARE nurse checks for each new resident admission if the resident has an advance care plan. |
|  |  |  |
| The leadership team decides who is responsible in the NH to guide the ACP process. |  |  |
| For residents in unstable condition before weekends: physician orders and care plans are clarified (Notfallplan), by the appointed responsible person(s) in each NH. |  |  |
| **Evidence-based tools** | | |
| **STOP & WATCH** | | |
| The INTERCARE nurse is responsible for the implementation of the STOP&WATCH and supervises the usage of the Stop and Watch STOP&WATCH tool in daily practice. | None | Degree of penetration of the STOP&WATCH tool, e.g., used by housekeeping staff, therapists.  Internal process of how the tools are handled and stored after completion.  Implementation of other tools such as care pathways, to help guide assessment for chronic conditions.  Using the tools to hand over information non-verbally, e.g., emails, fax. |
| Implementation of the STOP&WATCH tool on each participating unit, within the first 6 months of implementation of the model. |  |  |
| Used by nurse assistants to inform the responsible person about changes in resident condition. |  |  |
| It is clearly defined who will use the STOP&WATCH tool, if extended to other staff. |  |  |
| All staff using the STOP&WATCH must be trained. |  |  |
| The situation for which the STOP&WATCH tool is used, is recorded in the resident's documentation, if a change in resident situation has been recognized. |  |  |
| The nurse responsible should perform the adequate assessment after being given the STOP&WATCH. |  |  |
| The transmission of the STOP&WATCH tool is either indirect (e.g., storage in a designated compartment for the person in charge of the day) or it is handed over directly to the person in charge of the day / the responsible qualified nurse. |  |  |
| The STOP&WATCH tool must be filled in and, if necessary, the appropriate letters should be marked as soon as a change in the residents’ condition has been identified. |  |  |
| General information about the resident and the person who filled in the instrument must be added. |  |  |
| All unit staff are informed about implementation of the STOP&WATCH tool. |  |  |
| Distribution of the STOP&WATCH notepads to all employees who will use the tool. |  |  |
| **ISBAR** | | |
| The INTERCARE nurse is responsible for the implementation and monitoring of the use of ISBAR and in giving feedback. | None | None |
| Implementation of the ISBAR tool on each participating unit within the first 6 months of implementation of the model. |  |  |
| Used by registered nurses in communicating with physicians and with the INTERCARE nurse in acute situations. |  |  |
| It is clearly defined who will use the ISBAR tool, if extended to the members of the care team. |  |  |
| All staff using the ISBAR tool must be trained. |  |  |
| Distribution of the ISBAR Pocket version to all registered nurses and all staff trained to use the ISBAR tool. |  |  |
| All unit staff is informed about implementation of the ISBAR tool. |  |  |
| **Data-driven quality improvement** | | |
| Continuous data collection for all hospitalisations and emergency department visits, with exports every 3 months for statistical processing control (SPC) charts and 6 months for benchmarking. |  | Each NH can decide who participates in the SPC/Benchmarking discussion.  Each NH can decide who takes part in the discussion and completing one PDCA cycle |
| A member of the leadership team with or without/ INTERCARE nurse should discuss the SPC charts and benchmarking reports together and prepare discussion points for leadership meetings with the research group. |  |  |
| A member of the leadership team and INTERCARE nurse should meet and discuss which steps are needed to improve quality improvement and complete one PDCA cycle for one identified quality indicator. | For an identified issue, a Plan-Do-Check-Act cycle is carried out.  Plan  Pre-defined persons should think about how they will analyse a situation, how information will be collected, what the goal of the planned change is.  Do  Pre-defined persons should think about how they plan to carry out the change, what is needed, from whom, and who is responsible for guiding the change.  Check  Pre-defined persons should reflect on what was initially planned and what happened during the change.  Act  Pre-defined persons should discuss and describe which improvement measures were implemented and if change occurred. |  |

1. NH: Nursing home [↑](#footnote-ref-1)
2. RN: Registered nurse [↑](#footnote-ref-2)
3. BSN: Bachelor of Science in Nursing [↑](#footnote-ref-3)
4. MSN: Master of Science in Nursing [↑](#footnote-ref-4)
5. MAS: Master of advanced studies [↑](#footnote-ref-5)
6. HöFa: Bachelor of Nursing Science (BSN); higher education in nursing, levels I and II (HöFa I and II); Master of Nursing Science (MSN) Master of Advanced Studies (MAS); Registered Nurse (RN). [↑](#footnote-ref-6)
