# Supplementary material for: Evaluating the implementation fidelity to a successful nurse-led model (INTERCARE) which reduced nursing home unplanned hospitalisations
Source: BMC Health Serv Res. 2023 Feb 9;23:138. doi: 10.1186/s12913-023-09146-8 (PMC9910256; doi:10.1186/s12913-023-09146-8)
Supplement: Supplementary file 2 — Supplementary Material 2 [file 12913_2023_9146_MOESM2_ESM.docx]

**Additional file 2: Implementation strategies used to promote the up-take of INTERCARE**

**Implementation strategies**: The “how to” component of changing healthcare practice or the means and

methods of adopting and sustaining interventions, 8 strategies.

Summary of implementation strategies used to support and facilitate the implementation of the core elements of the intervention.

| **Implementation strategy** | **Implementation strategy and definition according to Powell et al^1^** | **Description for INTERCARE** |
| --- | --- | --- |
| **International and national nursing home visits** | Visit other sites  Capture local knowledge from implementation sites on how implementers and clinicians made something work in their setting and then share it with other sites. | During a preliminary phase A of the INTERCARE project, 15 case studies were conducted in Swiss NHs to assess structures, processes, outcomes as well as barriers and facilitators to facilitate the implementation and planned strategies to reduce barriers and ensure the sustainability of the intervention. International models were also visited to help gain an insight into ANP roles and model differences. |
| **Stakeholder meetings** | Conduct local consensus discussion  Include local providers and other stakeholders in discussions that address whether the chosen problem is important and whether the clinical innovation to address it is appropriate. | A stakeholder group formed of nursing home leaders, physicians, Swiss policymakers and cantonal association representatives, are included in important decisions regarding the intervention, such as decision making regarding the appropriateness of the clinical tasks and responsibilities of the new nurse expert role, defining the core elements of the intervention and to help identify barriers and facilitators for the implementation of the intervention, as well as discussing the outcomes for the Swiss setting.  Bi-annual meetings to exchange and discuss major points relating to the intervention. |
| **Binding contract between NHs and research site** | Obtain formal commitments  Obtain written commitments from key partners that state what they will do to implement the innovation. | A signed contract was established between the research site and the participating NHs. |
| **Determining core and peripheral components of the nurse-led model of care** | Promote adaptability  Identify the ways a clinical innovation can be tailored to meet local needs and clarify which elements of the innovation must be maintained to preserve fidelity. | The model consists of 6 core components and peripheral elements which allows the intervention to be tailored to meet the specific intervention site needs. Core components are considered to be mandatory to be implemented and peripheral elements can be adapted individually. Core components were developed and described to reach the specific clinical outcomes of the study. |
| **Nursing home leadership training and support** | Assess for readiness and identify barriers and facilitators  Assess various aspects of an organization to determine its degree of readiness to implement; barriers that may impede implementation, and strengths that can be used in the implementation effort. | Specifically, tailored training sessions for NH leadership and additional staff such as NH accountants, physicians and nurses to ensure buy-in and tailoring of the nurse-led model to individual NHs through the identification of barriers and facilitators. 1 full day and 2 half-day follow-up training sessions were offered to all 11 NHs participating. |
| **INTERCARE nurse blended learning curriculum** | Create new clinical teams  Change who serves on the clinical team, adding different disciplines and different skills to make it more likely that the clinical innovation is delivered (or is more successfully delivered) | Implementation of the INTERCARE nurses acquires new competencies and skills expanding the usual profile. Thus, position profile was developed and new competencies were described to ensure the ability to deliver the intervention. |
|  | Conduct ongoing training  Plan for and conduct training in clinical innovation in an ongoing way | Continuous education of INTERCARE nurses started before the project and was further developed throughout the project. |
|  | Resource sharing agreements  Develop partnerships with organizations that have the resources needed to implement the innovation | Partnerships with nursing educational institutions who have geriatric expertise and/or experience in curriculum development. |
|  | Make training dynamic  Vary the information delivery methods to cater to different learning styles and work contexts, and shape the training in the innovation to be interactive | Blended learning curriculum including: e-learnings, readings, tests, reflections and case studies and face-to-face meeting accounts for variation in delivering the education. It maximizes the learning outcomes considering that adults have different learning styles and working environments. |
|  | Develop and distribute educational materials  Distribute educational materials (including guidelines, manuals, and toolkits) in person, by mail, and/or electronically | Various materials as, e.g., guidelines on how to implement evidence-based tools, algorithms how and when to use Reflection tools, staff handouts to inform and power point presentations to educate staff about the communication instruments, manuals on how to enter residents' data into data management system, were developed and distributed. All materials should help facilitate the implementation and adherence to the intervention. All materials were posted on an online learning platform and/ or sent by email. |
| **Data collection for benchmarking and internal quality control** | Audit and provide feedback  Collect and summarize clinical performance data over a specified period and give it to clinicians and administrators to monitor, evaluate, and modify provider behaviour. | Quarterly exports for quality indicators and on-going collection of data for hospitalisations to help NHs identify where better quality of care can be provided and which actions they may take. The exports were discussed during the 2 monthly meetings in each NH. |
| **Continuous support of NH** | Provide local technical assistance  Develop and use a system to deliver technical assistance focused on implementation issues using local personnel | Project coordinator was available to provide assistance and ensure good communication between NHs and the research team.  Face-to-face two monthly meetings with the leadership teams. |
|  | Provide ongoing consultation  Provide ongoing consultation with one or more experts in the strategies used to support implementing the innovation | A networking platform was available for NHs to share experiences and documentation, as well as 2 monthly in-person meetings and 2 weekly phone calls to support the INTERCARE nurse during the implementation process. |

1. Powell BJ, Waltz TJ, Chinman MJ, et al. A refined compilation of implementation strategies: results from the Expert Recommendations for Implementing Change (ERIC) project. *Implement Sci* 2015;10:21. doi: 10.1186/s13012-015-0209-1 [published Online First: 2015/04/19]
