# Supplementary material for: Evaluating the implementation fidelity to a successful nurse-led model (INTERCARE) which reduced nursing home unplanned hospitalisations
Source: BMC Health Serv Res. 2023 Feb 9;23:138. doi: 10.1186/s12913-023-09146-8 (PMC9910256; doi:10.1186/s12913-023-09146-8)
Supplement: Supplementary file 3 — Supplementary Material 3 [file 12913_2023_9146_MOESM3_ESM.docx]

**Additional file 3: Fidelity measurement questionnaire**

| **Core component** | Yes (it is performed) | No (it is not performed) | Notes |
| --- | --- | --- | --- |
| **Interprofessional collaboration** | | | |
| A structure in place to facilitate interprofessional communication (e.g., meetings) between at least two different professions. |  |  |  |
| Noticing a resident issue and liaising with the relevant health care professional to establish the residents’ care goal. |  |  |  |
| Interpretation of assessment results and formulation of a resident care plan in collaboration with a member of the health care team. |  |  |  |
| The INTERCARE nurse supports the communication process between physicians and health care staff. |  |  |  |
| **INTERCARE nurse** | | | |
| According to the INTERCARE nurse’s skills and expertise residents are assessed in acute situations, when called by a member of the care team. |  |  |  |
| The INTERCARE nurse provides coaching to care staff on daily resident bedside needs. |  |  |  |
| The INTERCARE nurse plans educational sessions with care staff regularly. |  |  |  |
| The INTERCARE nurse drives team reflections for each reflection tool filled in. |  |  |  |
| The INTERCARE nurse must have 3 years-experience in long-term-care. |  |  |  |
| A position of 60% minimum per 80 beds for which the INTERCARE nurses are responsible for. |  |  |  |
| **Comprehensive geriatric assessment (CGA)** | | | |
| The INTERCARE nurse collaborates with the leadership and/or interprofessional team to discuss and define which assessment instrument they work with, for each of the 5 CGA dimensions in their institution, within the first 6 months of the implementation of the model. |  |  |  |
| The INTERCARE nurse’s role is clearly defined with regards to their input in the 5 dimensions of CGA. |  |  |  |
| The INTERCARE nurse is involved and supports the care team in integrating the 5 dimensions of CGA in daily practice. |  |  |  |
| The INTERCARE nurse ensures that residents and relatives are involved in the decision-making process. |  |  |  |
| **Advance care planning (ACP)** | | | |
| For every newly admitted resident, the following points must be documented in the residents’ records:   - - Do not resuscitate order   - Do not hospitalize order   - Use of antibiotics |  |  |  |
| The leadership team decides who is responsible in the NH to guide the ACP process. |  |  |  |
| For residents in unstable condition before weekends: physician orders and care plans are clarified (Notfallplan), by the appointed responsible person(s) in each NH. |  |  |  |
| **Evidence-based tools** | | | |
| **STOP & WATCH** | | | |
| The INTERCARE nurse is responsible for the implementation of the STOP&WATCH and supervises the usage of the Stop and Watch STOP&WATCH tool in daily practice. |  |  |  |
| Implementation of the STOP&WATCH tool on each participating unit, within the first 6 months of implementation of the model. |  |  |  |
| Used by nurse assistants to inform the responsible person about changes in resident condition. |  |  |  |
| It is clearly defined who will use the STOP&WATCH tool, if extended to other staff. |  |  |  |
| All staff using the STOP&WATCH must be trained. |  |  |  |
| The situation for which the STOP&WATCH tool is used, is recorded in the resident's documentation, if a change in resident situation has been recognized. |  |  |  |
| The nurse responsible should perform the adequate assessment after being given the STOP&WATCH. |  |  |  |
| The transmission of the STOP&WATCH tool is either indirect (e.g. storage in a designated compartment for the person in charge of the day) or it is handed over directly to the person in charge of the day / the responsible qualified nurse. |  |  |  |
| The STOP&WATCH tool must be filled in and, if necessary, the appropriate letters should be marked as soon as a change in the residents’ condition has been identified. |  |  |  |
| General information about the resident and the person who filled in the instrument must be added. |  |  |  |
| All unit staff are informed about implementation of the STOP&WATCH tool. |  |  |  |
| Distribution of the STOP&WATCH notepads to all employees who will use the tool. |  |  |  |
| **ISBAR** | | | |
| The INTERCARE nurse is responsible for the implementation and monitoring of the use of ISBAR and in giving feedback. |  |  |  |
| Implementation of the ISBAR tool on each participating unit within the first 6 months of implementation of the model. |  |  |  |
| Used by registered nurses in communicating with physicians and with the INTERCARE nurse in acute situations. |  |  |  |
| It is clearly defined who will use the ISBAR tool, if extended to the members of the care team. |  |  |  |
| All staff using the ISBAR tool must be trained. |  |  |  |
| Distribution of the ISBAR Pocket version to all registered nurses and all staff trained to use the ISBAR tool. |  |  |  |
| All unit staff is informed about implementation of the ISBAR tool. |  |  |  |
| **Data-driven quality improvement** | | | |
| Continuous data collection for all hospitalisations and emergency department (ED) visits, with exports every 3 months for SPC charts and 6 months for benchmarking. |  |  |  |
| A member of the leadership team with or without/ INTERCARE nurse should discuss the SPC charts and benchmarking reports together and prepare discussion points for leadership meetings with the research group. |  |  |  |
| A member of the leadership team and INTERCARE nurse should meet and discuss which steps are needed to improve quality improvement and complete one PDCA cycle for one identified quality indicator. |  |  |  |
