# Supplementary material for: Evaluating the implementation fidelity to a successful nurse-led model (INTERCARE) which reduced nursing home unplanned hospitalisations
Source: BMC Health Serv Res. 2023 Feb 9;23:138. doi: 10.1186/s12913-023-09146-8 (PMC9910256; doi:10.1186/s12913-023-09146-8)
Supplement: Supplementary file 4 — Supplementary Material 4 [file 12913_2023_9146_MOESM4_ESM.docx]

**Additional file 4**: Codes and definition as applied in the qualitative notes

| **Core element** | **Themes: Modifiable factors** | **Codes** | **Definition of codes** |
| --- | --- | --- | --- |
| **Interprofessional collaboration** | **Context** | Working practices | Organization of day to day “business” in the NH^[[1]](#footnote-1)^ |
|  |  | Cantonal policies | Policies in place for medical care delivery |
|  | **Participant responsiveness to the intervention’s delivery** | Changes in communication structures | Instances of working together from two or more different disciplines |
|  |  | Changes in practices | Impact / outcome in terms of changes to working practices |
|  |  | Resistance to change | Demonstration of difficulties to obtain buy-in from NH collaborators |
|  |  | Professional role | Perception of own or other’s roles, including empowerment (or lack thereof), professional pride) |
|  | **Quality of delivery** | Optimization in the communication process | Description of how the communication is facilitated in the NH |
|  |  | Results produced | End products of successful collaborations |
| **INTERCARE nurse** | **Context** | Existing role | Embedding the IN role in a prior existing structure supporting advanced nursing |
|  | **Participant responsiveness** | Role development within NH | Reactions of NH collaborators towards the IN'^[[2]](#footnote-2)^s role in the NH including evolvement of duties, role awareness, embeddedness of role in the NH |
|  |  | Reactions to the IN new role | Identified advantages, benefits, barriers, gaps of the IN role |
|  |  | IN role outcome | Skills, abilities, training and competencies, carried out or developed by the IN as discussed/as intended |
|  | **Strategies to facilitate implementation** | Avoiding negativity | Putting the emphasis on INTERCARE and IN scope to improve the future |
|  |  | High reachability | Actions to promote visibility, take part in unit activities |
|  | **Quality of delivery** | Commitment to the role | Extent to which efforts are invested in the IN role in the NH |
|  |  | Challenges to the delivery of IN role | Barriers which impede IN tasks and competencies |
| **Comprehensive geriatric assessment (CGA)** | **Context** | Contextual barriers | Contextual obstacles believed to hinder CGA^[[3]](#footnote-3)^ implementation |
|  | **Participant responsiveness** | Attitudes towards CGA | Way of thinking, feeling or perceiving the implementation of CGA in NHs |
|  | **Strategies to facilitate implementation** | Prioritizing to reduce burden | Actions taken to postpone the introduction of CGA and related tasks |
|  |  | Individual coaching | Support given on a peer to peer basis |
|  | **Quality of delivery** | Applying knowledge gained | Demonstration of conducting CGA or evidence of trying |
|  |  | Lack of follow-up | Description of a lack of follow-up after CGA is performed |
| **Advance care planning (ACP)** | **Context** | Expected progress | Development of ACP with regards to pre-implementation, processes, adaptions over time |
|  |  | ACP public theme | Generating interest as a theme which receives attention at present |
|  | **Participant responsiveness** | Medical engagement | Involvement (or non-involvement) of medical profession in the ACP^[[4]](#footnote-4)^ component, through support, praise, sharing discussions, paperwork |
|  | **Strategies to facilitate implementation** | Procedure to cover out-of-hours | System in place to anticipate issues occurring out of working hours or the will/ need to develop this |
|  |  | Providing document support | Description of documentation to help introduce, support, inform the introduction of ACP in NHs |

| **Core element** | **Themes: Modifiable factors** | **Codes** | **Definition of codes** |
| --- | --- | --- | --- |
| **Stop & Watch**  **ISBAR** | **Context** | Timeliness with resources | Missing needed materials |
|  | **Participant responsiveness** | Modes of usage | How information in the tools is used shared, integration of tools as electronic support, adaptations to them |
|  |  | Changes in communication practices | Perceived changes in communication within NHs and impact on practices |
|  |  | Attitudes towards the communication tools | Expressed feelings, opinions, self-efficiency about the communication tools |
|  |  | Perceived difficulties | Any challenges regarding the implementation, sustainment and usage of the communication tools |
|  | **Strategies to facilitate implementation** | Part of a routine | Integration in routine documentation delivered to new staff |
|  |  | Champions | Dedicated person which helps introduce the tools, can be unit or NH level |
|  |  | Pacing introduction | A progressive approach to implementation, statements of conscious non-implementation to secure implementation elsewhere |
|  |  | Enhancing visibility | Accounts of purposeful display on units |
| **Data driven quality improvement (DDQI)** | **Context** | Prior usage of DDQI^[[5]](#footnote-5)^ | A pre-study initiative to work with QI^[[6]](#footnote-6)^s |
|  |  | Responsibility level for DDQI | Description of how accountability for DDQI is organized in the NH |
|  | **Participant responsiveness** | Collaborative vision | Teamwork to obtain a result |
|  |  | Identified challenges for DDQI | Description of possible general barriers to conducting DDQI work |
|  |  | PDCA^[[7]](#footnote-7)^ application/responsiveness | Accounts given on the usage or adaptations to PDCA |
|  | **Strategies to facilitate implementation** | Enhancing understanding of QI | A way of providing support, guidance, information for the NHs to better understand DDQI |

1. NH: Nursing home [↑](#footnote-ref-1)
2. IN: INTERCARE nurse [↑](#footnote-ref-2)
3. CGA: Comprehensive geriatric assessment [↑](#footnote-ref-3)
4. ACP: Advance care planning [↑](#footnote-ref-4)
5. DDQI: Data-driven quality improvement [↑](#footnote-ref-5)
6. QI: Quality improvement [↑](#footnote-ref-6)
7. PDCA: Plan-Do-Check-Act [↑](#footnote-ref-7)
